# Mesenchymal Stromal Cells Derived from the Bone Marrow of Acute Lymphoblastic Leukemia Patients Show Altered BMP4 Production: Correlations with the Course of Disease

**DOI:** 10.1371/journal.pone.0084496

**Published:** 2014-01-06

**Authors:** Ángeles Vicente López, Miriam Nohemí Vázquez García, Gustavo J. Melen, Ana Entrena Martínez, Isabel Cubillo Moreno, Javier García-Castro, Manuel Ramírez Orellana, Agustín Gregorio Zapata González

**Affiliations:** 1 Department of Cell Biology, School of Medicine, Complutense University, Madrid, Spain; 2 Department of Oncohematology, Hospital Niño Jesús, Madrid, Spain; 3 Cellular Biotechnology Unit, Institute for Health Carlos III, Majadahonda, Madrid, Spain; 4 Department of Cell Biology, Faculty of Biology, Complutense University, Madrid, Spain; Wake Forest Institute for Regenerative Medicine, United States of America

## Abstract

The relevance of tumor microenvironment for the development and progression of tumor cells in hematological malignancies has been extensively reported. Identification of factors involved in the information exchange between the malignant cells and the bone marrow mesenchymal stem cells (BM-MSCs) and the knowledge on their functioning may provide important information to eliminate leukemic cells from protective BM niches. We evaluated changes in BM-MSCs obtained from children with acute lymphoblastic leukemia (ALL) at different times in the course of disease. Whereas ALL-MSCs did not exhibit phenotypic changes compared to BM-derived MSCs isolated from healthy donors, they exhibited increased adipogenic capacity. In addition, the viability of healthy CD34+ hematopoietic progenitors was significantly reduced when co-cultured with ALL-MSCs. ALL-MSCs grow less efficiently, although gradually recover normal growth with treatment. Accordingly, proliferation is particularly low in MSCs obtained at diagnosis and in the first days of treatment (+15 days), recovering to control levels after 35 days of treatment. Correlating these results with bone morphogenetic protein 4 (BMP4) production, a molecule demonstrated to affect MSC biology, we found higher production of BMP4 in ALL-MSCs derived from patients over the course of disease but not in those free of leukemia. However, no significant differences in the expression of different members of the BMP4 signaling pathway were observed. Furthermore, an inverse correlation between high levels of BMP4 production in the cultures and MSC proliferation was found, as observed in MSCs derived from patients at diagnosis that produce high BMP4 levels. In addition, co-culturing ALL-MSC with the REH leukemia cell line, but not CD34+ hematopoietic progenitors, powerfully enhanced BMP4 production, suggesting an intimate crosstalk among ALL-MSCs isolated from BM colonized by ALL cells that presumably also occurs in situ conditions. Our data may support the participation of BMP4 in BM niche, but the mechanism remains to be elucidated.

## Introduction

Bone marrow (BM) microenvironments are involved in the initiation and propagation of hematological diseases [1], [2]. It has been proposed that leukemia cells “hijack” the homeostatic mechanisms of the normal BM microenvironment in a process that becomes key for the response to chemotherapy and disease relapse [3]. Mesenchymal stromal cells (MSCs) are now recognized as the essential element of both healthy and leukemic hematopoietic microenvironments [4]. MSCs were first described as a BM-derived mononuclear cell fraction that, after ex vivo culture, adheres to plastic, acquires a fibroblast-like morphology [5], exhibits a non-hematopoietic phenotype, and shows capacity to differentiate into multiple mesodermal cell lineages [6]. Their role in hematological disorders has been particularly emphasized, but most of our knowledge of these topics comes from xenograft models, where cancer cells grow in non-physiological conditions, or using cell culture models where MSC are derived from healthy adult donors or even MSC are commercial lines. Little is known regarding the features of MSCs in cancer pediatric patients specifically in patients suffering acute lymphoblastic leukemia, the most common cancer diagnosed in children.

Many soluble and membrane-bound molecules have been related with the information exchange between malignant cells and BM-MSCs. In recent years, several studies have reported the relevance of BM stromal cells for the survival [7] and resistance to chemotherapy [8] of acute lymphoblastic leukemia (ALL) cells homed in the BM. These studies emphasized the relevance of cell-to-cell contacts between BM stromal cells and leukemia cells [7], [8] and the possible role played by certain molecules, such as IL7 [9], CXCR4 [10], and TGFβ [11]. In addition, bone morphogenetic proteins (BMPs), members of the TGFβ superfamily, and BM stroma are implicated in the development of hematopoietic neoplasms [12], [13], including ALL [14]. BMP6 released from BM stroma inhibits human B lymphopoiesis in adults [15], and BMP2 regulates MSC differentiation in humans. BMP4 has been described as a critical component produced by the hematopoietic microenvironment that regulates both HSC number and function [13] and recently Khurana et al have implicated BMP4 also in homing and engraftment of mouse and human hematopoietic stem/progenitor cells [16]. We recently demonstrated that MSCs derived from human adipose tissue endogenously produce BMP4, express all the molecular machinery of BMP4 signaling pathway, and respond in a concentration-dependent manner to the stimulation of this pathway [17]. In addition, in recent years the contribution of BMP4 to cancer pathogenesis has been emphasized reporting both protumoral and antitumoral effects of this morphogen, depending on the kind and level of risk of tumor [18]. Furthermore, BMP4 produced by tumor microenvironment seems to be important for the biology of numerous hematological [12], [19], and non-hematological tumors [20], [21], [22] although few studies have analyzed the cellular component responsible for such production, the autocrine effects or the stimulus responsible for this production.

In this study, we analyzed the behaviour of MSC in paediatric patients with acute lymphoblastic leukemia (ALL-MSCs), with special emphasis in the inverse correlation between high levels of BMP4 production in the cultures and MSC proliferation at diagnosis, during treatment and after remission (out of therapy), as well the relevance of leukemic cells in the powerfully enhanced BMP4 production of MSC at diagnosis and finally the important contribution of BMP4 in maintaining MSC in the hematopoietic niche. Our results indicate that BMP4 produced by BM stromal cells importantly influences on ALL cells.

## Materials and Methods

### Patients and samples

BM aspirates from eleven children diagnosed with B-cell precursor ALL at the Hospital del Niño Jesus (Madrid, Spain) were collected. The main characteristics of the patients in this study and the entire group are summarized in Table 1. BM samples were drawn at diagnosis (ALL-MSC-Diagnosis), and at 15 (ALL-MSC+15) and 35 days (ALL-MSC+35) after initiation of therapy; in some experiments, samples 52 and 70 days after therapy initiation (ALL-MSC+52, ALL-MSC+70) were used. Children were treated under the PETHEMA protocol (stands for Spanish Protocol for Malignant Hemopathies. Results of these protocols have been previously reported [23], [24]), and all samples after diagnosis were in complete haematological remission. Aspirates from three children with B-cell precursor ALL who recovered without signs of disease and out of therapy (OOT-MSC) (Table 1) and from six children with no haematological diseases (Healthy-MSC) were also obtained. The study was approved by the Ethics Committee of the Hospital del Niño Jesus and written informed consent was obtained from parents/tutors on the behalf of the minors/children participants.

**Table 1:** Clinic data of patients.

Patient	Sex	Age	ALL-Immunophenotype	Cytogenetics	Risk	Current Situation	Out of treatment
1	M	2	B-cell precursors	ND	L	In treatment-complete remission	NO
2	M	10	B-cell precursors	ND	L	3^rd^ year-complete remission	NO
3	M	10	B-cell precursors	ND	L	3^rd^ year-complete remission	NO
4	M	6	B-cell precursors	t(12;21)q(12;22)	L	2^nd^ year–complete remission	NO
5	F	2	B-cell precursors	ND	I	3^rd^ year-complete remission	NO
6	F	4	B-cell precursors	ND	I	2^nd^ year-complete remission	NO
7	F	5	B-cell precursors	ND	I	4^th^ year-complete remission	NO
8	M	5	B-cell precursors	ND	I	2^nd^ year-complete remission	NO
9	M	2	B-cell Precursors	45,XY,-20, del (1) (p31)	I	Intra-treatment relapse	NO
10	F	1	B-cell precursors	t(4;11)q(21;23)	H	Intra-treatment relapse and death	NO
11	M	14	B-cell precursors	t(9;22)q(4;11)	H	Intra-treatment relapse	NO
A	F	6	B-cell precursors	ND	I	4^th^ year-complete remission	1-year end of treatment follow up sample
B	F	9	B-cell precursors	t(12;21)q(12;22)	I	4^th^ year-complete remission	1-year end of treatment follow up the sample
C	M	10	B-cell precursors	ND	L	4^th^ year-complete remission	1-year end of treatment follow up sample

Note: M: male; F: female; ND: no determined; L: Low risk; I: Intermediate risk; H: High risk.

### Cord blood samples and leukemic cell lines

Cord blood Lin-CD34+ precursor cells were isolated using the CD34 Progenitor Cell Isolation Kit Miltenyi Biotech, followed by a negative selection to eliminate Lin+ cells.

The ALL cell lines were obtained from DSMZ (German collections of Microorganisms and Cell Culture) REH (ACC-22) and NALM6 (ACC128) both are B-cell precursor leukemia. The cell lines were maintained at a density of 1×10^6^ cells/mL in RPMI-1640 (GIBCO) in 10% FBS with antibiotics.

### Cell culture

MSCs were isolated and cultured as previously described [17], [25]. Once the cultures reached 80–90% confluence, the cells were recovered by supplying 0.25% trypsin solution (Gibco-Invitrogen) and counted in a hemocytometer. Cell viability was assessed by trypan blue staining, and the cells were replated at a density of 5×10^3^ cells/cm^2^. The cells were maintained and expanded in MesenPRO-RS™ medium (Gibco-Invitrogen), with antibiotics and L-glutamine in a 5% CO_2_-in-air incubator at 37°C. All experiments were performed with cells harvested between the fifth and tenth passage.

### Flow cytometry

The following mAbs conjugated with FITC, PE or APC were used for flow cytometric analysis: CD14, CD19, CD29, CD34, CD44, CD45, CD90, CD105 and HLA-DR (BD Biosciences, BioLegend, and Immunostep). For intracellular staining of phosphorylated SMAD1, cells were treated with Cytofix/Cytoperm solution (BD Biosciences), washed with Perm/Wash buffer III (BD Biosciences) and stained with anti-human phospho-Smad1/5/8 (Santa Cruz), followed by fluorochrome-conjugated multi-absorbed F(ab')2 fragment of donkey anti-rabbit IgG (Jackson ImmunoResearch Laboratories). Analyses were conducted in a FACSCalibur flow cytometer (BD Biosciences) at the Centro de Citometría y Microscopía de Fluorescencia (Complutense University-Madrid-Spain) (CCMF-UCM) and a FACS Canto II (BD Bioscience) with the FACS Diva software, at the Hospital Niño Jesus.

### Proliferation assays

Cultures were pulsed for 12 hours with 10 µM BrdU. A BrdU Labeling and Detection Kit III (ROCHE) were used to measure BrdU incorporation into newly synthesized DNA, as described previously [17].

### Apoptosis assays

Cells were stained with Annexin-V-FITC (ROCHE) and propidium iodide, and analyzed by flow cytometry. Annexin-V-positive and propidium iodide-negative cells were considered apoptotic cells [17].

### BMP4 measurements

The concentration of BMP4 in the culture supernatants was determined using a specific ELISA assay kit (R & D Systems) [17], following the manufacturer's instructions.

### BMP4 and dorsomorphin treatment

Heathy-MSC and ALL-MSC-diagnosis were cultured for six days in Mesen PRO-RS™ medium supplemented with human recombinant BMP4 (0.01–100 ng/mL). For treatment with dorsomorphin (Calbiochem, Nothingham), cells were cultured in Mesen PRO-RS™ medium for 24 h and then the medium was supplemented with the inhibitor (2–10 µM) for five more days. After treatment, MSCs were harvested and used for viability assays or processed for determining cell proliferation.

### PCR analysis

Real-time PCR reactions were performed with specific TaqMan Gene Expression Assays (Applied Biosystems), as described previously [17]. Expression was normalized using GNB2L1 and calculated based on CT values [17]. Amplifications, detections, and analyses were performed in a 7.900HT Fast Real-time PCR System (Centro de Genómica, Complutense University, Madrid, Spain).

### Differentiation assays

The procedures were adapted from previous protocols [25]. BM-MSCs were plated at a cell density of 5000 cells/cm^2^ and grown for six days in Mesen PRO-RS™ medium. The cells were then cultured with adipogenic or osteogenic differentiation medium [17]. After six days of differentiation, the cells were collected for quantitative PCR analysis. After fifteen to twenty days, lipid and ALP quantification was performed as previously described [26], [27].

### Co-culture assays

ALL-MSCs were co-cultured with either CD34+ cells purified from cord blood units using immunomagnetic methods (Miltenyi Biotech) or the B-precursor leukemic cell line REH (ratio MSC:CD34+ and MSC:REH, 1∶20). Viability of cells was determined separately for hematopoietic cells (identified as CD45+ cells) and MSCs (CD105+ cells) by flow cytometry after five days in culture.

### Statistical analysis

The Mann-Whitney (Wilcoxon) test and Student *t* test were used for statistical analysis. *P≤0.05, **P≤0.01.

## Results

### Immunophenotype of leukemic and healthy MSCs

**Figure 1:**
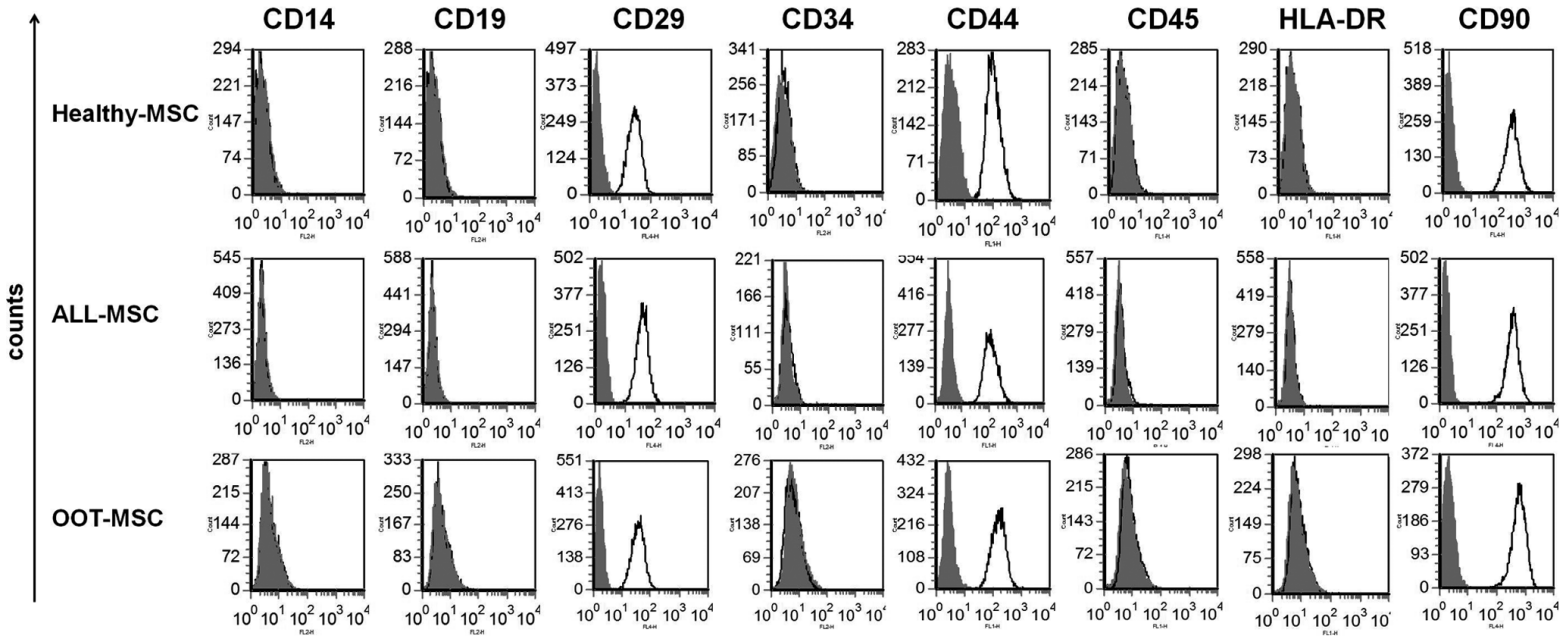
Surface marker expression pattern of MSCs derived from BM of ALL patients. No significant changes were observed in the expression of molecules between Healthy-MSC, ALL-MSC and OOT-MSC. Gray histograms represent background fluorescence using isotype-matched irrelevant mAbs.

Healthy-MSCs, OOT-MSCs and ALL-MSCs expressed typical MSC markers, including CD29, CD44, and CD90, but no hematopoietic cell markers (CD14, CD19, CD34 or CD45) or the MHC class II molecules, including HLA-DR (Fig. 1). No significant changes were detected in the expression of studied markers among the different groups of MSCs.

### Growth kinetics of leukemic and healthy MSCs

**Figure 2:**
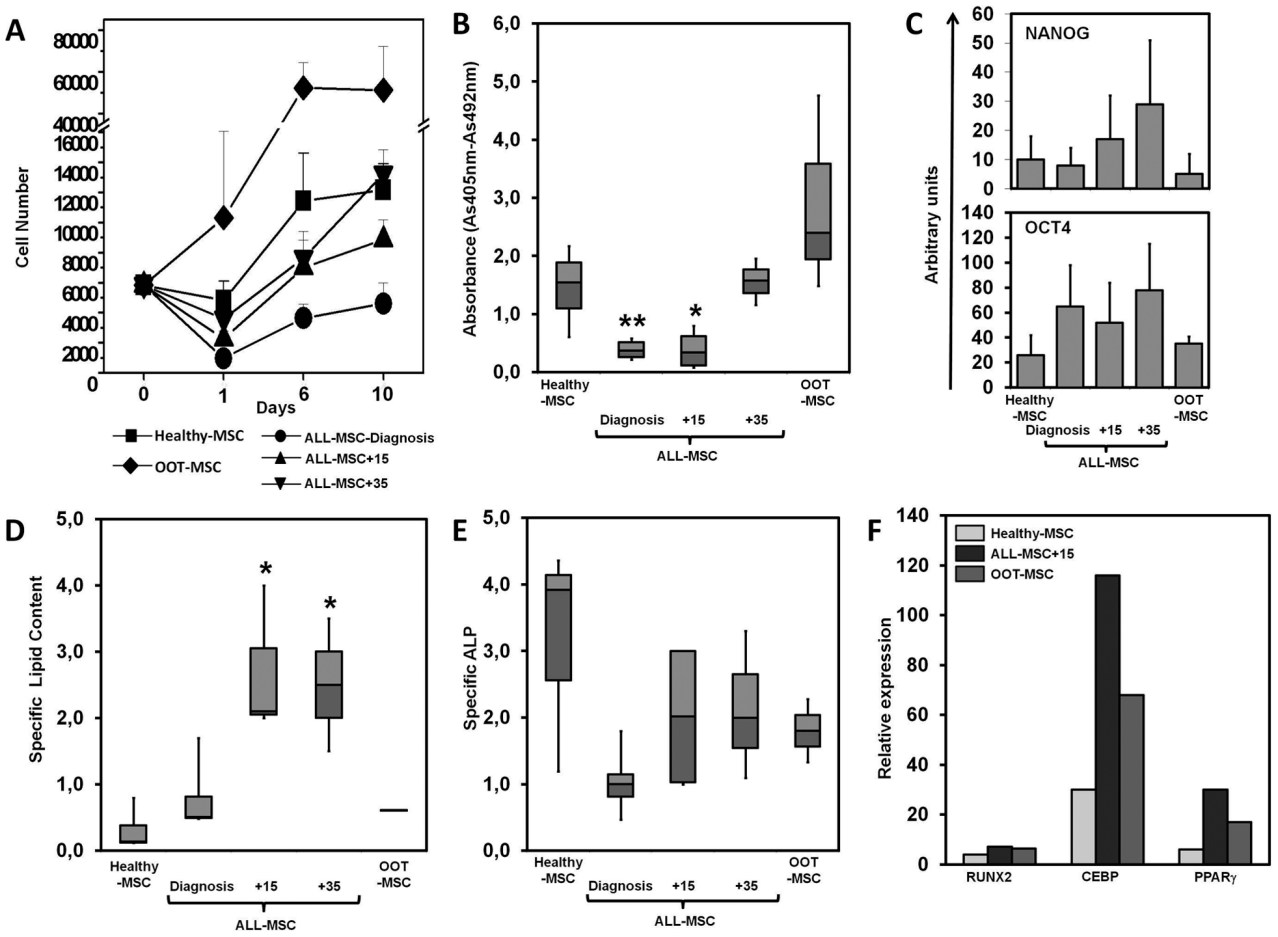
Growth kinetics, proliferation and differentiation of ALL-MSCs. (A) Growth kinetics of BM-MSCs. At each time point, cells were harvested, stained with trypan blue, and counted. Viable cells are represented. Data are representative results from at least, three independent experiments. (B) Determination of BM-MSC proliferation after culture for six days. Results are the mean of 11 different donors for ALL-MSCs and 6 different donors for Healthy-MSCs, each with three cultures per point. Asterisks indicate statistical significance between Healthy-MSC and ALL-MSC-Diagnosis or ALL-MSC+15 (*P<0.05; **P<0.01). (C) Expression of transcription factors essential to the pluripotency and self-renewal properties of undifferentiated stem cells. MSCs cultured for six days were collected and the expression of NANOG and OCT4 was analyzed by quantitative PCR. No significant statistical differences were observed between Healthy-MSCs and ALL-MSCs. (D–F) Osteogenic and adipogenic differentiation capacity of Healthy-MSCs and ALL-MSCs. After six days of culture in Mesen PRO-RS™ medium, cells were transferred to either osteoblast- or adipocyte-inducing media for 15–20 more days. Adipogenic differentiation was evaluated by Oil Red O (ORO) staining of neutral lipids and Coomassie brilliant blue staining of cellular protein S. The specific lipid content was calculated as the absorbance ratio obtained by ORO and Coomassie blue staining. Osteogenic differentiation was evaluated as alkaline phosphatase (ALP) activity in 50 µL of cell lysate and normalized for total protein to obtain the specific ALP. Asterisks indicate statistical significance between Healthy-MSC and ALL-MSC+15 or ALL-MSC+35 (*P<0.05) (Healthy-MSC, n = 6; ALL-MSC, n = 11; OOT-MSC, n = 3). (F) After six days of culture in osteoblast- or adipocyte-inducing media, cells were collected and analyzed by quantitative PCR. Data show the induction of adipose (CEBP and PPARγ) and osteogenic (RUNX2) specific genes in different MSC samples.

Despite the high variability found among the samples, the growth pattern over 10 days was similar for Healthy-MSCs and ALL-MSCs (Fig. 2A). In both Healthy-MSCs and ALL-MSC cultures, an initial decrease of MSC numbers on day 1 was followed by a gradual increase at later time points. However, whereas ALL-MSC-Diagnosis and ALL-MSC+15 numbers did not recover to control levels, ALL-MSC+35 reached control values by day 10 of culture. OOT-MSC showed a robust growth throughout the culture (Fig. 2A). In agreement with the reduced MSC numbers found in ALL-MSC-Diagnosis and ALL-MSC+15 cultures (Fig. 2A), both showed a significant decrease in the proliferative rate compared with healthy levels (Fig. 2B). In contrast, ALL-MSC+35 showed similar proliferation levels as healthy cells (Fig. 2B). OOT-MSCs showed higher proliferation rates than healthy cells, although the differences were not statistically significant (Fig. 2B).

### Differentiation capabilities of healthy and leukemic MSC

In vitro capacity for adipogenic and osteogenic differentiation was evaluated in Healthy-MSCs and ALL-MSCs (Fig. 2C–F). Prior to differentiation, both ALL-MSCs and Healthy-MSCs expressed similar levels of NANOG and OCT4, two markers that confirmed the undifferentiated condition of the MSC groups (Fig. 2C). Whereas ALL-MSC-Diagnosis and OOT-MSC groups exhibited an adipogenic differentiation capacity similar to healthy cells, ALL-MSCs from treated patients (+15 and +35) showed an increased adipogenic differentiation compared to healthy cells (Fig. 2D). Concomitantly, we observed a higher expression of genes specifically involved in adipogenesis, CEBP and PPARγ, in these samples (Fig. 2F). In contrast, no significant differences were found in ALP expression in both ALL-MSCs and OOT-MSCs compared to Healthy-MSCs (Fig. 2E). Furthermore, the induced expression of the osteogenic gene RUNX2 was similar in all samples analyzed (Fig. 2F).

### ALL-MSCs differentially affect survival of healthy and leukemia cell progenitors

**Figure 3:**
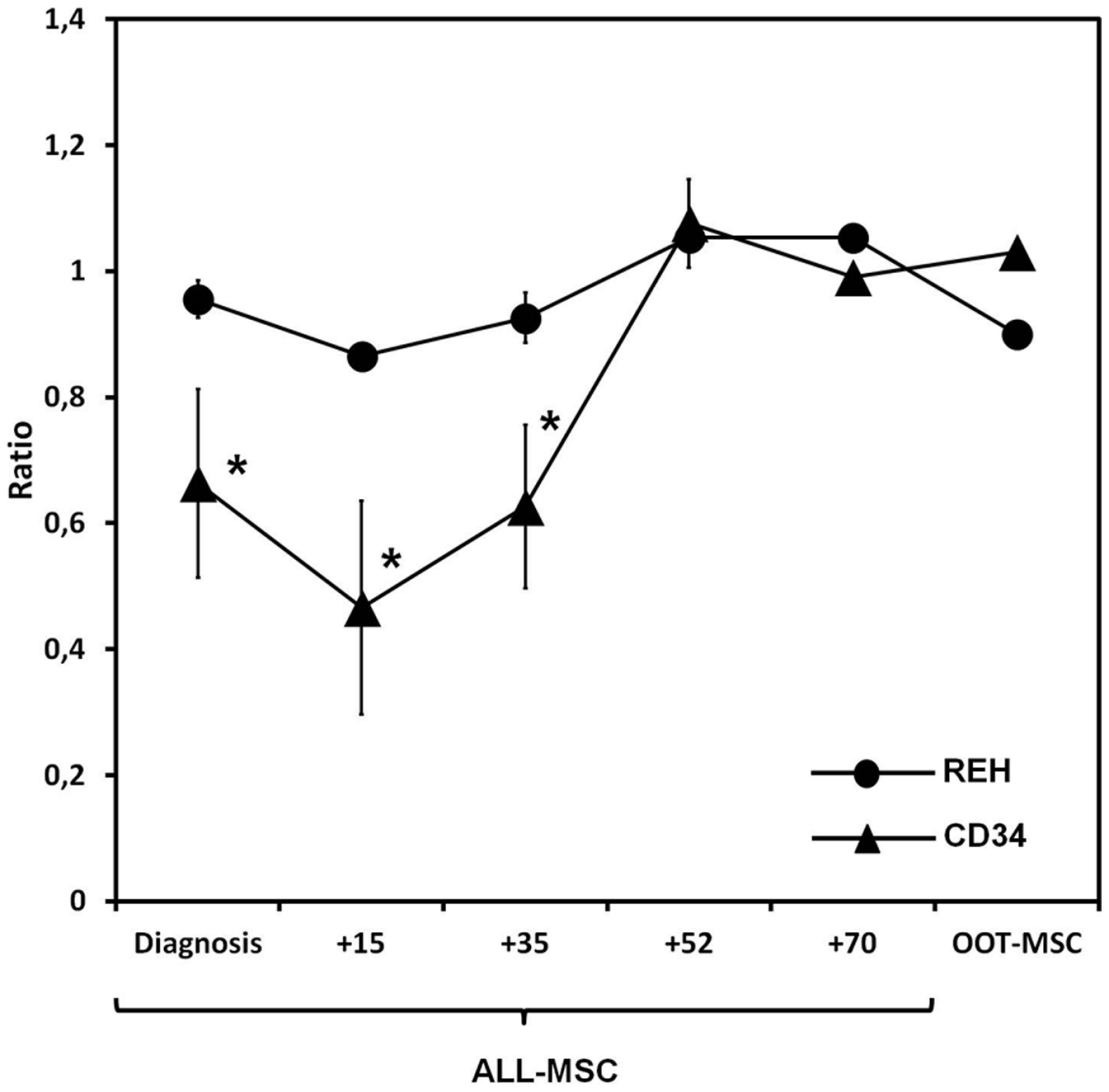
Survival of cord blood CD34+ hematopoietic progenitors and leukemic cells (REH cell-line) co-cultured with ALL-MSCs. Cell survival is expressed as the ratio between the survival of CD45+ cells from co-cultures with ALL-MSCs and that from co-culture of either hematopoietic or leukemia cells with Healthy-MSCs. Values below 1 indicate that the viability of hematopoietic cells was lower when co-cultured with ALL-MSCs than when co-cultured with healthy BM-MSCs. Note the significant decrease of the survival of CD34+ hematopoietic progenitors co-cultured with ALL-MSCs at diagnosis and after +15 and +35 days of treatment. (*P≤0.05).

Because controversial results have been published on the capacities of leukemia microenvironments for differentially supporting the growth of normal hematopoietic and leukemia cells [28], we evaluated the effects of ALL-MSCs on the survival of either cord blood CD34+ hematopoietic progenitors or REH, a leukemic cell line, by co-culturing both cell types with ALL-MSCs. The viability of CD45+ cells co-cultured with ALL-MSC was compared to healthy or leukemia cells co-cultured with Healthy-MSCs (Fig. 3). Co-culturing leukemia REH cells with ALL-MSCs did not affect REH cell viability compared to co-cultures with the Healthy-MSC controls. However, viability, was reduced significantly in healthy CD34+ hematopoietic progenitors co-cultured with MSCs derived from patients at the first stages of the disease (-Diagnosis, +15, +35, +52, +70) and OOT-MSC. The relative survival of healthy hematopoietic progenitors recovered normal values when co-cultured with ALL-MSCs derived from +52 patients (Fig. 3).

### BMP4 production by healthy and leukemic MSCs at distinct stages of the disease

**Figure 4:**
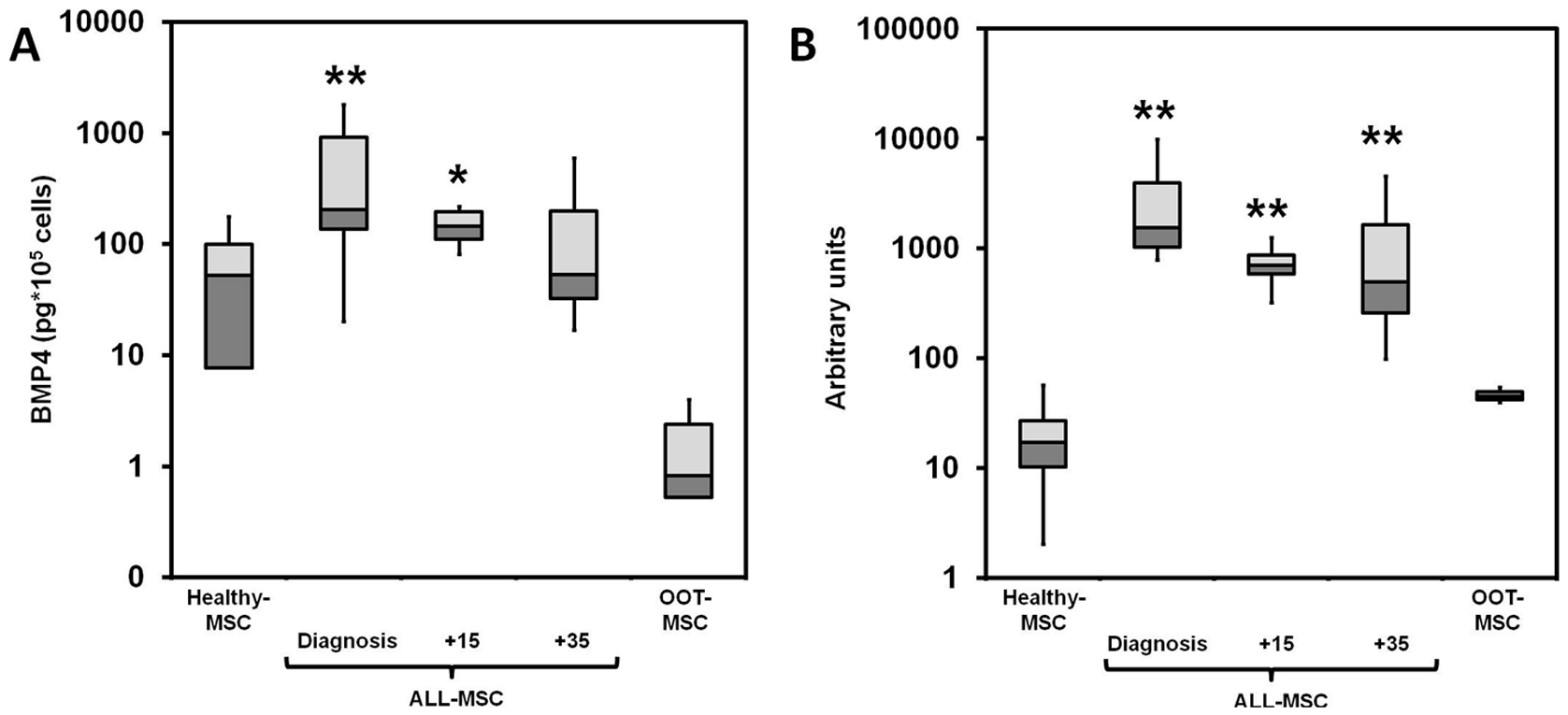
Expression of BMP4 by ALL-MSCs in the course of disease. (A) MSCs isolated from the BM of healthy donors (n = 6) and ALL patients at different times in the course of disease (n = 11 patients and n = 3 children out of therapy) were cultured for six days, and the amount of BMP4 in the medium was assessed by ELISA. (B) BMP4 transcript expression in Healthy-MSCs and ALL-MSCs was confirmed by quantitative PCR. Data represent arbitrary units of gene expression from the different samples studied. Asterisks indicate statistical significance between Healthy-MSCs and ALL-MSCs (*P≤0.05; **P≤0.01).

ALL-MSCs obtained at diagnosis showed 12-fold more BMP4 in the culture supernatants than Healthy-MSCs. After 15 days of treatment (+15), this difference was reduced to just two-fold, although in both cases the differences with control values were statistically significant. In contrast, BM-MSCs of patients after 35 days of treatment (+35) showed low levels of BMP4, similar to Healthy-MSCs (Fig. 4A). The observed differences in BMP4 transcript production between Healthy-MSCs and ALL-MSCs were even more significant (Fig. 4B). ALL-MSCs at diagnosis showed 166-fold more BMP4 transcripts than Healthy-MSCs. These values gradually decreased in ALL-MSC+15 and ALL-MSC+35, but remained significantly higher than control values (Fig. 4B). In contrast, levels of BMP4 transcripts in MSCs obtained from patients out of therapy did not exhibit significant differences compared to Healthy-MSCs (Fig. 4B).

### Expression of different members of the BMP4 signaling pathway in healthy and leukemic MSCs at different stages of disease

**Figure 5:**
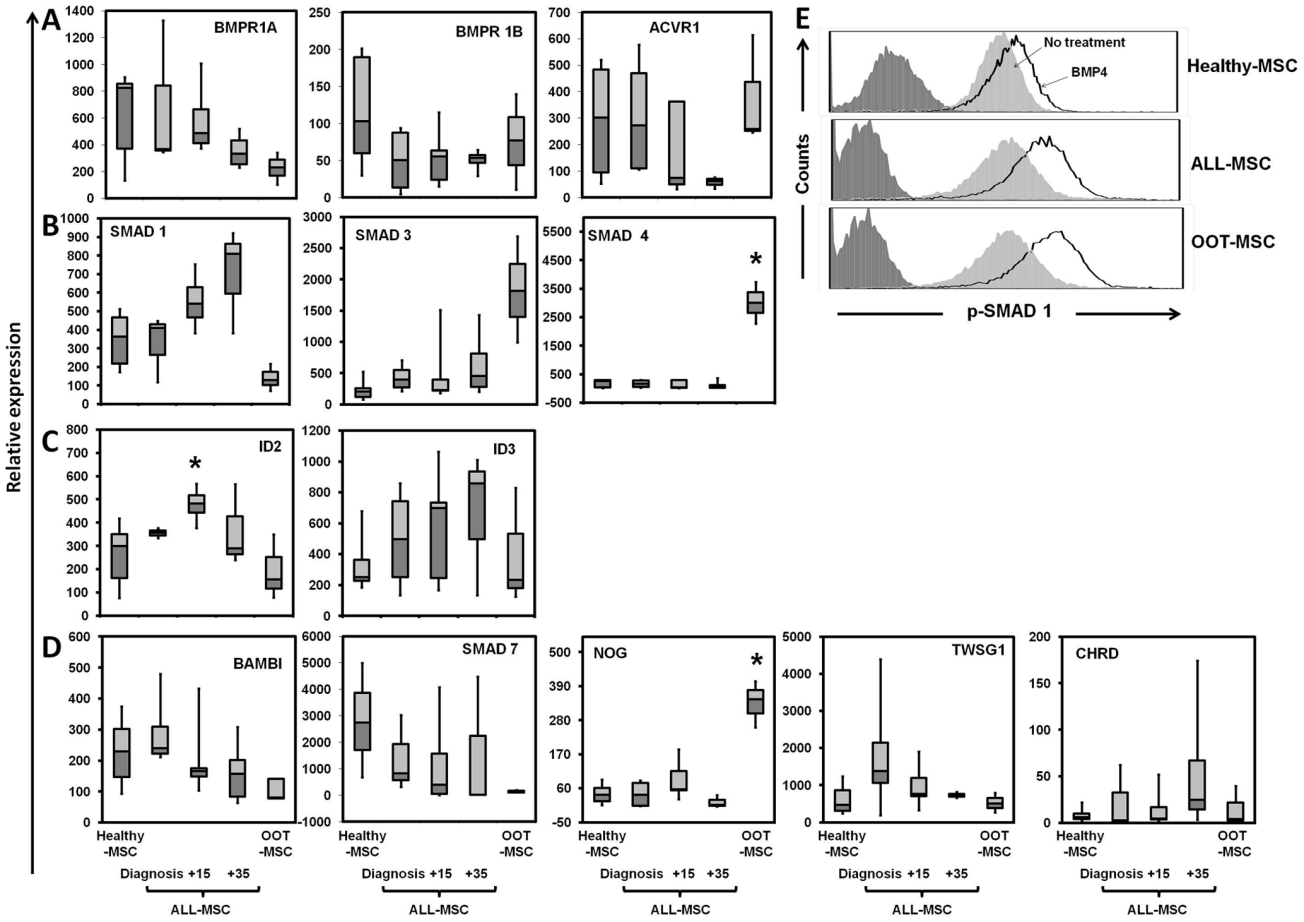
BMP2/4 signaling pathway in ALL-MSCs in the course of disease. Expression of different components of the BMP2/4 signaling pathway as determined by quantitative PCR: (A) receptors, (B) R-SMADs and Co-SMADs, (C) ID proteins and (D) inhibitors of BMP signaling. PCR data are represented as arbitrary units of gene expression. Asterisks indicate statistical significance between Healthy-MSCs (n = 6) and ALL-MSCs (n = 11) or OTT-MSCs (n = 3) (*P≤0.05). (E) MSCs were cultured for six days in Mesen PRO-RS™ medium alone (light gray histogram) or supplemented with BMP4 (100 ng/mL) (open histogram) for the last 30 minutes. Detection of intracellular SMAD1 phosphorylation was performed by flow cytometry. Background staining is shown by isotype-matched control Abs (dark gray histogram). The results are representative of three different experiments.

BMPs bind to three type I receptors (ACVR1, BMPR1A, and BMPR1B) and three type II receptors (BMPRII, ACVR2A, and ACVR2B). In our study, we focused on the type I receptors that show high affinity for the members of the BMP2/4 subgroup of BMPs [29]. In general, the expression of the three type I receptors was similar or lower in leukemic MSCs, including those from children out of therapy, but the differences were not significant (Fig. 5A).

After ligand binding a heterodimeric-activated receptor complex is formed, that phosphorylate R-SMADs, which then join to common SMAD4 and translocate into the nucleus where they affect ID genes [30]. Although a slight increase was detected in the expression of three R-SMADs in the leukemic cells at diagnosis, +15, +35 and out of therapy, the differences were not statistically significant. Remarkably, SMAD4 expression was significantly higher in MSCs obtained from BM of children after ending therapy compared to other MSC groups, including healthy ones (Fig. 5B). On the other hand, we were unable to find significant variations in the expression of the target transcription factors of BMP4, ID2 and ID3, between leukemic and Healthy-MSCs (Fig. 5C). ID2 expression was only significantly increased in ALL-MSC+15 (Fig. 5C).

Additionally, our results showed no changes in the expression of different BMP4 antagonists in ALL-MSCs compared to Healthy-MSCs, except in the MSCs isolated from BM out of therapy, which exhibited significantly higher Noggin expression than any other MSC groups, including healthy controls (Fig. 5D).

Furthermore, to determine whether the BMP signaling pathway was functional in ALL-MSCs, we stimulated healthy and ALL-MSCs with BMP4 and evaluated the phosphorylation of R-Smad1 by flow cytometry. Our results confirmed the normal functioning of the BMP signaling pathway in ALL-MSCs (Fig. 5E).

### Response of healthy and leukemic MSC to BMP4 pathway

**Figure 6:**
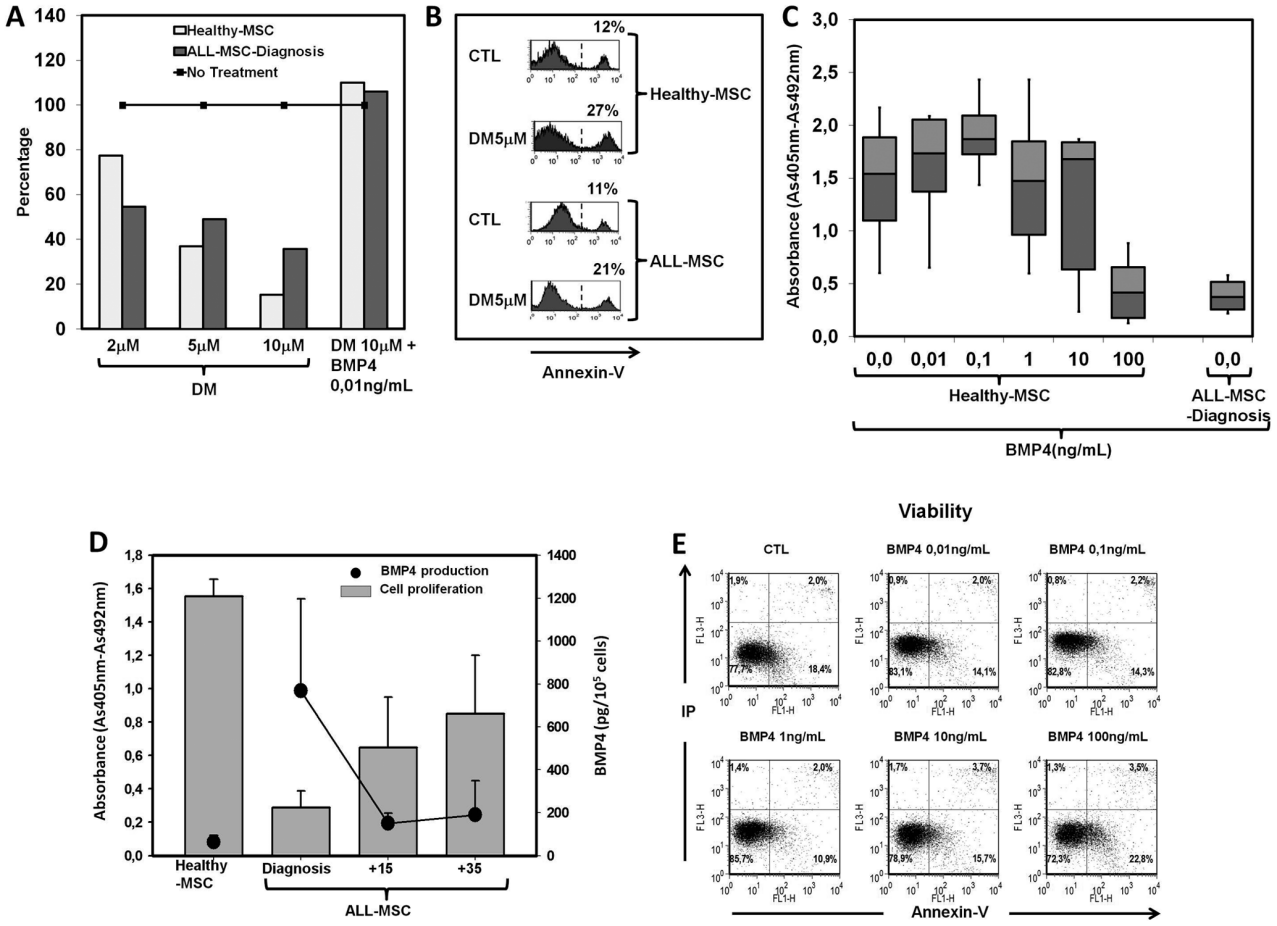
Relevance of BMP4 signaling in proliferation and viability in ALL-MSCs. (A) Effects of different doses of dorsomorphin, an inhibitor of the canonical BMP signaling pathway, on the viability and proliferation of Healthy-MSCs and ALL- MSCs. BM-MSCs were cultured for six days in Mesen PRO-RS™ medium alone (no treatment) or containing different doses of dorsomorphin. Cultures treated with dorsomorphin 10 µM were supplied with 0.01 ng/mL BMP4 to avoid non-specific effects of the drug. Data represent the percentage of proliferation of treated Healthy-MSC or ALL-MSC-Diagnosis cultures with respect to that of non-treated ones in five independent experiments. (B) Annexin V staining was measured by flow cytometry in Healthy-MSC and ALL-MSC-Diagnosis cells harvested from control and dorsomorphin-treated cultures on day 6. The percentages of Annexin V+ cells in the iodide-negative population are indicated in each histogram. Data are representative of three independent results. (C) Cell proliferation measured as BrdU incorporation in newly synthesized ADN of Healthy-MSCs cultured for six days in the presence of increasing concentrations of BMP4. Note the low proliferation in cultures treated with high doses of BMP4 (100 ng/mL) and its similarity to that found in non-treated cultures of ALL-MSC-Diagnosis endogenously producing high amounts of the morphogen. Data represent the mean ±SD of five independent experiments, each with three cultures per time point. (D) Correlations between the proliferative rate of Healthy-MSCs and ALL-MSCs at different times in the course of disease and BMP4 production assessed in the 6 day culture supernatants. Note the gradual increase of MSC proliferation in correlation with the decreased production of BMP4. (E) Viability of Healthy-MSCs treated six days with different doses of BMP4 analyzed by Annexin V and propidium iodide staining. Results are representative of three independent experiments.

Treatment with different doses of dorsomorphin, an inhibitor of the canonical BMP signaling pathway, resulted in a significant concentration-dependent reduction of MSC proliferation that was greater in Healthy-MSCs than in ALL-MSCs (Fig. 6A). BMP4 supply to dorsomorphin-treated cultures restored cell proliferation to control levels, eliminating possible non-specific effects of the dorsomorphin treatment (Fig. 6A). At the same time, increased proportions of apoptotic Healthy-MSCs and ALL-MSCs where detected when cultured with dorsomorphin (Fig. 6B).

The addition of increasing doses of BMP4 induced a slight increase of healthy MSC proliferation compared to non-treated Healthy-MSCs, whereas high doses of BMP4 significantly reduced proliferation (Fig. 6C). Remarkably, when high doses of BMP4 were added to the Healthy-MSC cultures, the levels of MSC proliferation were equivalent to those observed in ALL-MSCs at diagnosis (Fig. 6C), when the highest amounts of BMP4 are produced (Fig. 4). Thus, when we represented together BMP4 production and MSC proliferation in ALL-MSCs along the course of disease, a correlation could be established between the high production of BMP4 and the low levels of MSC proliferation at diagnosis, followed by a gradual decrease of BMP4 production during treatment (+15, +35) that correlated with increased rates of cell proliferation (Fig. 6D). On the other hand, none of the BMP4 doses significantly modified Healthy-MSC viability (Fig. 6E).

### BMP4 production in co-cultures of healthy and leukemic MSC with either cord blood CD34+ hematopoietic progenitor cells or REH leukemia cell line

**Figure 7:**
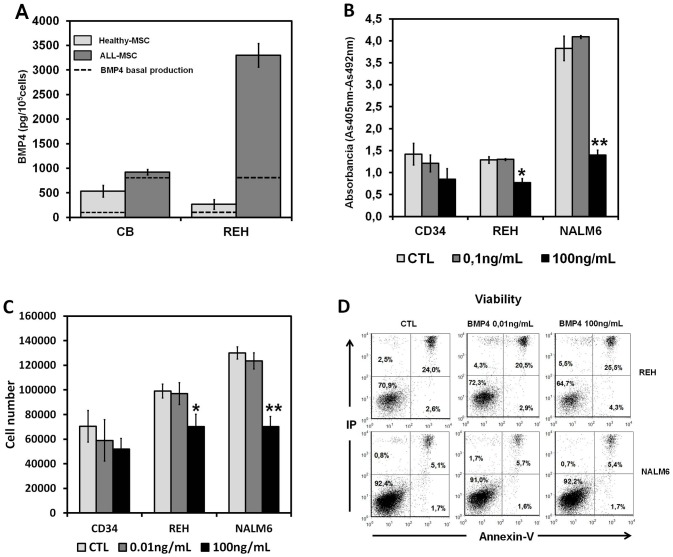
Leukemic cells induce BMP4 production by ALL-MSCs and are differentially affected by the morphogen. (A) BMP4 production was determined by ELISA assays in supernatants of 24 hours from BM-MSCs cultured alone or either with cord blood CD34+ hematopoietic progenitor cells or pre-B ALL cell line, REH. BMP4 basal production (dotted line) in Healthy-MSCs (gray) and ALL-MSCs (dark gray) (ALL-MSC-Diagnosis) cultures increased after co-culture with cord blood CD34+ hematopoietic progenitors or with REH cells, respectively, but not when healthy progenitors were co-cultured with ALL-MSCs or REH cells with Healthy-MSCs. Data represent the mean ±SD of three experiments for each group. (B) Proliferative rate of different leukemic cell lines and cord blood CD34+ cells cultured without MSCs in the presence of distinct doses of BMP4. The figure shows BrdU incorporation after six days of culture (*P≤0.05). (C). Proliferation of different leukemic cells lines and cord blood CD34+ cells, cultured without MSC in the presence of distinct dosed of BMP4. The figure shows cell numbers after culture. Results are the mean of three experiments. Asterisks indicate statistical significance (*P<0.05; **P<0.01). (D) Percentages of apoptotic cells in both REH and NALM6 leukemic cell lines in the presence of different doses of BMP4.

Because a crosstalk between stroma (MSCs) and hematopoietic cells has been established, we evaluated the effect of the presence of hematopoietic progenitors or leukemic cells (REH cell line) on BMP4 production by Healthy-MSCs or ALL-MSCs. When co-cultures were established between healthy or leukemic MSCs and CD34+ hematopoietic cells, although the basal BMP4 production of ALL-MSCs alone was significantly higher (767 pg±480) than that of Healthy-MSCs (62 pg±42) (Fig. 7A, dotted line), the presence of hematopoietic progenitors further stimulated production in the co-cultures established with Healthy-MSCs than in those with ALL-MSCs. However, co-cultures established with the REH cell line plus ALL-MSCs, but not with Healthy-MSCs, dramatically increased BMP4 production (Fig. 7A).

To analyze possible effects of this increased production of BMP4 by ALL-MSCs on the growth of either healthy or leukemic hematopoietic cells, we studied the proliferative capacity and viability in different leukemic cell lines and healthy hematopoietic cells, cultured alone without MSC, in the presence of high and low amounts of BMP4. No changes were observed in the proliferation rate in cultures supplied with low doses of BMP4. In contrast, high doses induced a slight decrease of the percentage of proliferating cells that was significant in the case of both REH cells and NALM6 cells (Fig. 7B) and in correlation, the absolute numbers obtained in these cultures were also reduced (Fig 7C). BMP4 treatment did not significantly affect the proportions of apoptotic REH and NALM6 leukemic cells (Fig. 7D).

## Discussion

ALL-MSCs do not show phenotypic changes compared to healthy BM-MSCs but their capacity to differentiate to adipocytes increases during the course of disease, later partially regressing in MSCs derived from patients who ended the therapy. Presumably, the chemotherapy treatments affect the behaviour of MSCs increasing their capacities for adipogenic differentiation because cells derived from bone marrow of patients without treatment (at diagnosis and out of treatment) exhibited the same capacity than those derived from healthy donors. Other authors have reported that BMP4 is involved in MSC commitment into adipocytes [31] in a process in which FAK (focal adhesion kinase) plays an important role [32]. However, in MSC derived from ALL treated patients other factors apart from BMP4 production should be involved because ALL-MSC at different stages of disease produce similar amounts of BMP4 (Fig 4A and 4B) but exhibit different adipogenic capacities. In addition, their pattern of growth and proliferative capacities are altered during the disease. In general, no phenotypic changes have been reported in MSCs isolated from patients with haematological malignancies [33], [34]. Nevertheless, some studies reported decreased expression of some markers [35], [36]. Data in the literature are also contradictory with respect to differentiation capacities: some studies have reported normal osteogenic, chondrogenic and adipogenic differentiating potential in leukemic MSCs [34], whereas others have shown impaired chondrogenesis or adipogenesis [33], [37]. Different protocols for evaluating the differentiating properties of MSC could explain these different results.

Remarkably, our results demonstrate changes in growth and proliferation of MSCs during the course of the disease and after complete remission, which presumably correlated with the altered production of BMP4 by BM-MSCs isolated from patients in distinct stages of the disease. In this regard, a significant decrease was detected in MSC proliferation in samples obtained at diagnosis or after 15 days of treatment, whereas ALL-MSC+35 reached healthy values on day 10 of culture, and MSCs from patients free of therapy grow more than the Healthy-MSCs, perhaps reflecting a rebound effect after the end of treatment. In agreement, other authors have reported defective proliferation capacity of MSCs derived from leukemia and myelodysplasic syndrome patients [34], [37].

Many molecules affect the biology of MSCs and some appear to be altered in different pathologies, but it is difficult to establish a comprehensive notion of their true role. Our results show a significant increase in BMP4 production by MSCs from patients at diagnosis that gradually decreases during treatment, and in MSCs derived from patients who had ended treatment. BMP4, a member of the TGFβ superfamily that regulates cellular survival, proliferation and differentiation as well as cell fate determination [38], has been reported as critical for the normal hematopoietic microenvironment [13], [39]. BMP was also shown to be involved in numerous hematopoietic neoplasias [12], [40] and other non-haematological tumours [20], [21], [22], [41]. Although high doses of BMP4 reduce proliferation of some leukemia cell lines, the effects of BMP4 could be mainly mediated by the stroma components [42] rather than directly on hematopoietic cells. We and other authors have previously demonstrated effects on the MSC biology of healthy donors [17], [43], and primary and immortalized cell lines derived from human BM stroma inhibit the proliferation of ALL cell lines in a process blocked by Noggin, a BMP antagonist [11].

However our results did not detect significant modifications in the expression of BMP signaling pathway components. This could reflect the high variability detected among samples, probably due of differences in features in the group of patients such as age, sex, cytogenetic characteristics or level of risk of disease. Furthermore, the lack of changes in the BMP4 canonical pathway does not exclude the activation of other intimately associated intracellular pathways, particularly in the case of MSCs derived from individuals who had finished therapy. In this group, co-SMAD4 expression was increased but not expression of BR-SMADs 1, 5 and 8. However, SMAD3, which is principally activated by activin and TGFβ rather than BMP4, also increases. In children out of therapy, this canonical signaling pathway could have been activated by other ligands. In addition, MSCs derived from children after finishing therapy show increased Noggin expression that could be antagonizing BMP4 effects and promoting disease relapse. Alternatively, this increase may reflect the recovery of a marrow free of leukemia after months of chemotherapy. Nevertheless, it is a speculative explanation that needs further confirmation.

The involvement of BMP4 in the altered behaviour of leukemia MSC is evidenced also by the responses observed to the different doses of the morphogen and to the blockade of the signaling pathway. As observed in human adipose stem cell cultures [17], treatment with the BMP inhibitor dorsomorphin resulted in a dose-dependent decrease in cell proliferation that was greater in healthy MSCs compared to leukemia MSCs, even when high doses of dorsomorphin were used. This finding is presumably related to the higher production of BMP4 found in leukemia MSCs, compared to that of healthy cultures. More remarkable are the results obtained after healthy MSCs cultures were treated with different doses of BMP4. In agreement with our previous results [17], MSCs derived from normal BM showed decreased proliferation and viability when high concentrations of BMP4 were added to the cultures. Other results support this relationship between BMPs and MSC behaviour. Thus, the Noggin-dependent decreased endogenous production of BMP reduces the human BM-MSC survival [43] and high doses of BMP2 reduce MSC growth [44]. Accordingly, MSCs derived from ALL patients at diagnosis that produce endogenously high amounts of BMP4 exhibit low levels of proliferation that gradually increase during treatment in correlation with decreased BMP4 production.

We also analyzed the relevance of leukemia cells for BMP4 production by MSCs. Remarkably, whereas normal CD34+ hematopoietic progenitors favoured the BMP4 production by Healthy-MSCs but hardly affected that of ALL-MSCs, the co-culture of ALL-MSCs with the ALL REH cell line amplified their BMP4 production. Hence, our results support a true crosstalk between ALL cells and the altered BM microenvironment. ALL cells could “hijack” the homeostatic mechanisms that govern the BM hematopoietic microenvironment, as previously proposed by other authors [3]. Underlying mechanisms that govern the effects of leukemia cells on BMP4 genes in BM-MSCs are still unknown, although some ideas can be proposed. Sonic hedgehog (SHH) proteins could be key in this activation. Paracrine requirement for Shh signaling is observed in B-cell malignancies and other cancers [45], [46], [47]. Different BMPs and SHH proteins participate in the control and differentiation of human hematopoietic precursor cells, together with several cytokines [39], [48], [49], [50]. BMPs mediate SHH activity in many systems during organogenesis and in adult life. BMP4 acts as a downstream mediator of SHH-dependent effects on intrathymic CD34+ precursor cells, whereas SHH up regulates BMP receptor expression and induces autocrine BMP4 secretion on CD34+ cells. In addition, Noggin reverses the inhibition of CD34+ cell proliferation induced by Shh [51] Other cytokines, such as IL3, IL6, FLT3 ligand, SCF and G-CSF, up regulate BMP4 expression in human cord blood CD34+ CD38- precursor cells[42] and the combination of IFN-γ, IL17, TNF-α and TGFβ strongly induces the production of BMP2 by human MSCs [52].

How the altered behaviour of ALL-MSCs could affect the biology of leukemic and hematopoietic cells; and whether those presumptive effects are mediated through the high production of BMP4 found in ALL-MSC cultures remain unknown. ALL-MSC-Diagnosis decreases the survival of healthy hematopoietic progenitors compared to that observed in co-cultures with BM-MSC from healthy donors, but does not substantially modify survival of leukemic cells. Other studies have reported impaired haematopoiesis in the presence of MSCs derived from patients suffering distinct haematological syndromes [37], [53] and the occurrence of leukemia cells in the BM disrupts hematopoietic stroma causing abnormal behaviour of healthy hematopoietic progenitors [3].

Furthermore, we demonstrate that high levels of BMP4 reduce, although not significantly, the proliferation of healthy hematopoietic progenitors, but particularly that of the leukemic REH and NALM6 cell lines. Presumably, in the microenvironment of a bone marrow homed by leukemic cells, high production of BMP4 could directly affect the ALL-MSCs and then indirectly the hematopoietic progenitors. Thus, decreased survival of healthy CD34+ progenitor cells occurs in co-cultures with ALL-MSCs that exhibit a high capacity to differentiate to adipocytes. Remarkably, MSCs that undergo adipogenic differentiation protect acute myeloid leukaemia from retinoid acid-induced death [54] but adipocytes negatively affect normal haematopoiesis in the BM [55].
